# Practice Patterns of Fundoscopic Examination for Diabetic Retinopathy Screening in Primary Care

**DOI:** 10.1001/jamanetworkopen.2022.18753

**Published:** 2022-06-27

**Authors:** Ailin Song, Jay B. Lusk, Kyung-Min Roh, Kevin J. Jackson, Karen A. Scherr, Ryan P. McNabb, Ranee Chatterjee, Anthony N. Kuo

**Affiliations:** 1 Duke University School of Medicine, Durham, North Carolina; 2 Duke University Fuqua School of Business, Durham, North Carolina; 3 Department of Ophthalmology, Duke University, Durham, North Carolina; 4 Department of Family Medicine and Community Health, Duke University, Durham, North Carolina; 5 Department of Medicine, Duke University, Durham, North Carolina; 6 Department of Biomedical Engineering, Duke University, Durham, North Carolina

## Abstract

**Importance:**

Primary care professionals (PCPs) have a central role in screening for diabetic retinopathy (DR), especially in settings where access to specialty eye care is limited. Data on current DR screening practice patterns in primary care are needed to inform screening strategies.

**Objectives:**

To assess the practice patterns of fundoscopic examination for DR screening in a large primary care network and to evaluate the sensitivity and accuracy of PCP fundoscopy for detecting DR.

**Design, Setting, and Participants:**

A retrospective cohort study was performed using random sampling and manual review of electronic health records of PCP fundoscopic examination documentation compared with documentation of an examination performed by an eye care professional (ophthalmologist or optometrist) within 2 years before or after primary care encounters. From a single-institution primary care network of 28 clinics, 7449 adult patients with diabetes seen at least once in the primary care network in 2019 were eligible for this study. Data from 2001 encounters were abstracted from the electronic health record for a random sample of 767 patients. Data analysis was performed from January 2021 to May 2022.

**Exposure:**

Fundoscopic examination by PCPs.

**Main Outcomes and Measures:**

The frequency of PCPs performing fundoscopy at least once in the calendar year for patients with diabetes. Univariate and multivariable logistic regression analyses were performed to identify patient, clinician, and clinic factors associated with PCPs performing fundoscopy at least once in the calendar year. The PCP examination results were compared with diagnoses made by eye care professionals to assess the sensitivity and accuracy of the findings from PCP examinations.

**Results:**

Among the 767 adult patients with diabetes included in the analysis, 387 (50.5%) were female, and the median age was 64 years (IQR, 54-71 years). Primary care professionals documented a fundoscopic examination for 93 patients (12.1%); all results were documented as normal. When eye care professional examination results were used as the reference standard, the accuracy of PCP fundoscopic examination was 62.7% (95% CI, 50.0%-73.9%) and sensitivity for detecting disease was 0.0% (95% CI, 0.0%-14.9%). No patient demographic or clinical characteristics were associated with PCPs performing fundoscopy. In multivariable logistic regression, the number of PCP years in practice was associated with greater odds of patients receiving fundoscopy at least once in the year (adjusted odds ratio per 10 years in practice, 1.26; 95% CI, 1.01-1.59; *P* = .04); having nurse practitioner credentials was associated with lower odds of receiving fundoscopy (adjusted odds ratio, 0.23; 95% CI, 0.04-0.79; *P* = .049; compared with having physician credentials); after adjusting for rural clinic location, clinic location in a primary care shortage area, and documentation of an up-to-date eye care professional examination by a PCP in the study year.

**Conclusions and Relevance:**

In this cohort study, fundoscopic examination was rarely performed and was not sensitive for detecting DR in primary care practice. Because the rate of DR screening by eye care professionals remains low, research to explore and break down barriers to the implementation of effective primary care–based DR screening strategies, such as teleretinal imaging, is needed to prevent vision loss from undiagnosed DR.

## Key Points

**Question:**

How frequently and accurately do primary care professionals (PCPs) perform fundoscopic examination to screen patients with diabetes for diabetic retinopathy in clinical practice?

**Findings:**

In this cohort study of 2001 encounters involving 767 adult patients with diabetes seen in a large primary care network, PCPs performed a documented fundoscopic examination for 12.1% of patients. The sensitivity of PCP-performed fundoscopy for detecting disease was 0.0%.

**Meaning:**

These findings suggest that fundoscopy may be a suboptimal method to screen for diabetic retinopathy in primary care; research to facilitate implementation of more effective primary care–based diabetic retinopathy screening strategies is needed.

## Teaser

This cohort study uses data from a large primary care network to assess the frequency and accuracy of fundoscopy for diabetic retinopathy screening performed by primary care professionals vs eye care professionals for adults with diabetes.

## Introduction

Diabetic retinopathy (DR) is the leading cause of blindness in working-age adults (aged 20-74 years) in the US.^1^ The American Academy of Ophthalmology^2^ and the American Diabetes Association^3^ recommend regular eye screenings performed by eye care professionals (ophthalmologists or optometrists) to prevent vision loss from DR. However, despite the use of myriad public health strategies to improve DR screening rates, such as full Medicare coverage for DR screening, at least 40% of patients in the United States do not receive recommended screenings for DR.^2,4^

Primary care professionals (PCPs)—defined as physicians, nurse practitioners, and physician assistants involved in the provision of primary care medical services—have a central role in DR screening, especially in settings where access to specialty eye care is limited.^5^ Previous studies using standardized training and testing protocols have demonstrated that fundoscopy can be an effective DR screening tool in primary care.^6,7^ However, limited time and resources^8^; insufficient confidence, knowledge, and examination skills^9^; and the general movement toward laboratory and imaging evaluation instead of physical diagnosis^10^ may create barriers to PCPs performing fundoscopy in clinical practice. Given the paucity of data on this topic, we conducted a retrospective cohort study to assess practice patterns of fundoscopic examination for DR screening in a large primary care network.

## Methods

This study was approved by the Duke University Institutional Review Board with an informed consent waiver because the study was a retrospective cohort analysis and posed no more than minimal risk to participants. This study followed the Strengthening the Reporting of Observational studies in Epidemiology (STROBE) reporting guidelines, and a STROBE checklist was provided.

All patients aged 18 years or older with type 1 or type 2 diabetes who were seen at least once at 1 of the 28 clinics within the Duke Health primary care network in 2019 were identified using the *International Classification of Diseases, Ninth Revision, Clinical Modification* and *International Statistical Classification of Diseases, Tenth Revision, Clinical Modification* codes listed in the eTable in the Supplement. A subset of patients was randomly selected for medical record review. All primary care encounters for diabetes care or general wellness during 2019 were manually reviewed for the cohort. Within each encounter, the patient’s most recent hemoglobin A_1c_ level, insulin status (ie, taking or not taking insulin), history of posterior eye disease, current eye concerns, and PCP documentation of fundoscopic examination were reviewed. Documentation by a PCP of whether patients had seen an eye care professional within 1 year before the PCP visit (eye examination up-to-date) was also abstracted. The eye care professional visit closest in date (either before or after the primary care encounter) to each primary care encounter, if within 2 years of the encounter, was also reviewed for diagnoses and examination findings. Patient age, sex, race, ethnicity, type of insurance, diabetes type, PCP, and primary care clinic visited were abstracted using the Duke Enterprise Data Unified Content Explorer.^11^ Primary care professional credential (physician, physician assistant, or nurse practitioner) and years in practice were abstracted from institutional provider profiles and the Federation of State Medical Boards database.^12^ Clinics were categorized as located or not located in rural areas, primary care shortage areas, and medically underserved areas based on classifications in the Rural Health Information Hub.^13^ Numbers of ophthalmologists per capita per clinic were obtained from county-level data from the Area Health Resources Files.^14^

### Statistical Analysis

#### Power Analysis

A sample size of 706 was required to have an 80% power at α = .05 to detect an odds ratio (OR) of 1.5 for a risk factor with a 1% event rate at the mean value of the risk factor in a multiple logistic regression model. A multiple correlation coefficient of 0.5 between the risk factor of interest and all other risk factors was used for the outcome of fundoscopy being performed at least once during the 2019 calendar year by the patient’s PCP.

#### Descriptive Statistics

Descriptive statistics were used to characterize the study population. Counts with percentages were provided to describe categorical data; medians with IQRs were used to summarize continuous variables, which were not normally distributed. Because proportions of missing data were low, complete case analysis was used.

#### Univariable Regression Models

Data were analyzed from January 2021 to May 2022. Univariate and multivariable logistic regression analyses were performed to identify patient, clinician, and clinic factors associated with PCPs performing fundoscopy at least once in the calendar year. Variables for which the CIs of univariable ORs did not cross 1 were included in the multivariable model. For univariable analyses, *P* values were not reported (CIs alone were reported). Confidence intervals for univariable patient-level logistic regression analyses were calculated using profile likelihood estimation. For encounter-level analyses, cluster-robust CIs were generated using the Wald method.

#### Calculation of Sensitivity and Accuracy

Eye care professional examination results were used as the reference standard. Primary care professional fundoscopic examination sensitivity for detecting disease was calculated as the proportion of true-positive results (patients with an abnormal finding on eye examination by a PCP and an abnormal finding on eye examination by an eye care professional) to the sum of false-negative results (patients with a normal finding on eye examination by a PCP and an abnormal finding on eye examination by an eye care professional) and true-positive results. Accuracy was calculated as the proportion of the sum of true results (true-positive and true-negative results) to all results (true-positive, false-positive, true-negative, and false-negative results). Confidence intervals for sensitivity and accuracy were calculated using the exact binomial method. Sensitivity analyses were conducted to address potential sources of bias.

#### Multivariable Logistic Regression Model

The full multivariable model was reported without additional variable selection. Continuous variables were assessed for the linearity of log-odds assumption, the Cook distance was used to identify influential points, and the variance inflation factor was calculated to evaluate for multicollinearity. Encounter-level logistic regression analyses used cluster-robust standard errors to control for multiple encounters for each patient. The threshold for statistical significance was set at α = .05 for 2-sided tests.

#### Statistical Software

Statistical analysis was performed in R, version 4.0.2 (R Foundation for Statistical Computing) with RStudio Desktop, version 1.2.5019. Logistic regression was performed using the R Stats package. Cluster-robust standard errors were calculated using the rms package.

## Results

A total of 7449 adult patients with diabetes who were seen at least once in the primary care network in 2019 were eligible for this study. A total of 2001 encounters from 767 randomly selected patients were included in the study. The median patient age was 64 years (IQR, 54-71 years); 387 (50.5%) of the patients were female, and 380 (49.5%) were male. Patient characteristics are provided in Table 1. Primary care professionals documented a fundoscopic examination for 93 patients (12.1%); all findings were documented as normal. A total of 422 patients (55.0%) had an eye care professional visit within 2 years of the PCP encounter. A total of 312 patients (40.1%) both did not have an eye care professional visit and did not receive a fundoscopic examination from their PCP. Diagnoses made during eye care professional visits are provided in Table 2.

**Table 1.** Characteristics of the Patient Cohort From 2001 Primary Care Encounters, Stratified by Receipt vs Nonreceipt of Fundoscopy at Least Once in 2019

Characteristic	Overall (N = 767)	Received ≥1 PCP fundoscopic examination		Odds ratio (95% CI)		*P* value^a^
		No (n = 674)	Yes (n = 93)	Unadjusted	Adjusted	
Age at first visit, median (IQR), y	64 (54-71)	64 (55-71)	60 (52-70)	0.86 (0.72-1.03)^b^	NA	NA
Sex, No. (%)						
Female	387 (50.5)	343 (50.9)	44 (47.3)	1 [Reference]	NA	NA
Male	380 (49.5)	331 (49.1)	49 (52.7)	1.15 (0.75-1.79)	NA	NA
Race, No (%)						
African American or Black	297 (38.7)	267 (39.6)	30 (32.3)	0.74 (0.46-1.19)	NA	NA
Alaska Native or American Indian	1 (0.1)	1 (0.2)	0	NA^c^	NA	NA
Asian	29 (3.8)	25 (3.7)	4 (4.3)	1.06 (0.30-2.86)	NA	NA
Native Hawaiian or Pacific Islander	2 (0.3)	1 (0.2)	1 (1.1)	NA^c^	NA	NA
White	404 (52.7)	351 (52.1)	53 (57.0)	1 [Reference]	NA	NA
≥1 Race	16 (2.1)	13 (1.9)	3 (3.2)	NA^c^	NA	NA
Other^d^	10 (1.3)	10 (1.5)	0	1.28 (0.46-3.02)	NA	NA
Not reported	8 (1.0)	6 (0.9)	2 (2.2)	NA^c^	NA	NA
Ethnicity, No (%)						
Hispanic	17 (2.2)	13 (1.9)	4 (4.3)	2.29 (0.63, 6.65)	NA	NA
Non-Hispanic	727 (94.8)	641 (95.1)	86 (92.5)	1 [Reference]	NA	NA
Not reported	23 (3.0)	20 (3.0)	3 (3.2)	1.12 (0.26-3.35)	NA	NA
Insurance type at first visit						
Commercial	361 (47.1)	309 (45.8)	52 (55.9)	1 [Reference]	NA	NA
Medicare Advantage	183 (23.9)	165 (24.5)	18 (19.4)	0.65 (0.36-1.12)	NA	NA
Medicare	199 (25.9)	180 (26.7)	19 (20.4)	0.63 (0.35-1.08)	NA	NA
Medicaid	19 (2.5)	16 (2.4)	3 (3.2)	1.11 (0.25-3.49)	NA	NA
Tricare	5 (0.7)	4 (0.6)	1 (1.1)	1.49 (0.08-10.3)	NA	NA
Yearly mean HbA_1c_ level, median (IQR), % of total Hb	6.83 (6.35-7.65)	6.83 (6.30-7.70)	6.85 (6.55-7.25)	0.93 (0.77-1.10)	NA	NA
Taking insulin at any point in 2019, No. (%)						
No	605 (78.9)	532 (78.9)	73 (78.5)	1 [Reference]	NA	NA
Yes	162 (21.1)	142 (21.1)	20 (21.5)	1.03 (0.59-1.71)	NA	NA
No. of encounters per patient, median (IQR)	2 (1-3)	2 (2-3)	2 (1-4)	1.01 (0.88-1.14)	NA	NA
Type of diabetes						
Type 1	30 (3.9)	27 (4.0)	3 (3.2)	0.80 (0.19-2.32)	NA	NA
Type 2	737 (96.1)	647 (96.0)	90 (96.8)	1 [Reference]	NA	NA
Eye concern reported at any PCP encounter, No. (%)	33 (4.3)	31 (4.6)	2 (2.2)	0.46 (0.07-1.54)	NA	NA
≥1 Eye care professional examination documented in 2019 by PCP						
Up-to-date	466 (60.8)	404 (59.9)	62 (66.7)	1 [Reference]	1 [Reference]	NA
Not up-to-date	156 (20.3)	145 (21.5)	11 (11.8)	0.94 (0.89-1.00)	0.56 (0.27-1.08)	.10
Not documented	145 (18.9)	125 (18.5)	20 (21.5)	1.00 (0.95-1.07)	0.97 (0.54-1.69)	.92

Abbreviations: HbA_1c_, hemoglobin A_1c_; NA, not applicable; PCP, primary care professional.

SI conversion factor: To convert HbA_1c_ to a proportion of total Hb, multiply by 0.01.

^a^ Values are from the multivariable model, which included years in practice of PCP; PCP credential; rural clinic location; whether eye care professional examination was documented as up-to-date, documented as not up-to-date, or not documented; and clinic location in a primary care shortage area.

^b^ Per 10 years of age.

^c^ Alaska Native or American Indian, more than 1 race, Native Hawaiian or Pacific Islander, and patients for whom race was not reported were combined with the Other group into a single group for regression analysis because group sizes were so small as to lead to issues with perfect separation with this covariate.

^d^ The Duke Enterprise Data Unified Content Explorer was used to collect patient racial and ethnic information, which was based on patient self-report. In that system, patients could self-identify as other.

**Table 2.** Eye Care Professional Visits and Diagnoses

	Patients, No. (%) (N = 767)	Received ≥1 PCP fundoscopic examination in 2019, No. (%)	
		No (n = 674)	Yes (n = 93)
Eye care professional visit ≤2 y before or after any primary care encounter	422 (55.0)	362 (53.7)	60 (64.5)
Eye care professional diagnosis			
Nonproliferative diabetic retinopathy	56 (13.3)	48 (13.3)	8 (13.3)
Proliferative diabetic retinopathy	12 (2.8)	12 (3.3)	0
Diabetic macular edema	15 (3.6)	13 (3.6)	2 (3.3)

Abbreviation: PCP, primary care professional.

A total of 24 PCPs (12.2%) performed fundoscopy for at least 1 patient; the median number of times PCPs performed fundoscopy during the year was 0 (IQR, 0-0 times; range, 0-25 times). For the 59 patients who had fundoscopic examinations performed by both a PCP and an eye care professional, PCP examination accuracy was 62.7% (95% CI, 50.0%-73.9%) and sensitivity for detecting disease was 0.0% (95% CI, 0.0%-14.9%) (Table 3).

**Table 3.** PCP vs Eye Care Professional Fundoscopy Results

PCP fundoscopic examination results^a^	Eye care professional fundoscopy results, No. (%)	
	Abnormal (n = 22)	Normal (n = 37)
Abnormal (n = 0)	0	0
Normal (n = 59)	22 (37.3)	37 (62.7)

Abbreviations: PCP, primary care professional.

^a^ Sensitivity: 0.0% (95% CI, 0.0%-14.9%); accuracy: 62.7% (95% CI, 50.0%-73.9%).

To evaluate whether results were biased by changes in patients’ clinical presentations between visits with a PCP and an eye care professional, we performed a sensitivity analysis restricting the cohort to the 232 patients who had an eye care professional visit within 3 months before or after any primary care visit. This analysis showed that 19 patients received a fundoscopic examination from their PCPs, with all results documented as normal by the PCPs; 9 of these patients (47.4%) had an abnormal finding when the examination was performed by an eye care professional (accuracy of PCP fundoscopy, 52.6%; 95% CI, 31.7%-72.7%; sensitivity for detecting disease, 0.0%; 95% CI, 0.0%-29.9%). An additional sensitivity analysis that limited the sample to only diabetes follow-up visits showed that 14 patients received fundoscopy at least once in the year; 10 saw an eye care professional within 2 years before or after any primary care visit. Results of fundoscopy for all 14 patients were documented by PCPs as normal; on examination by an eye care professional, 6 patients had normal findings and 4 had abnormal findings (accuracy, 60.0%; 95% CI, 31.3%-83.2%; sensitivity, 0.0%; 95% CI, 0.0%-49.0%).

Patient-level univariable logistic regression analyses showed that hemoglobin A_1c_ level, age at first encounter, insulin use, diabetes type, sex, race, ethnicity, insurance type, and report of an eye concern at any PCP encounter during the year were not associated with whether PCPs performed fundoscopy at least once in 2019 (Table 1). Clinic location in a medically underserved area and the number of ophthalmologists per capita were also not associated with whether PCPs performed fundoscopy at least once in 2019 (Table 4). The only patient-level factor included in the multivariable analysis was documentation by the patient’s PCP at least once in the year that an eye care professional examination was not up to date (OR, 0.94; 95% CI, 0.89-1.00 compared with having a documented up-to-date eye examination). Primary care professional and clinic factors included in the multivariable analysis were clinic location in a rural area (OR, 0.36; 95% CI, 0.20-0.61), clinic location in a primary care shortage area (OR, 0.43; 95% CI, 0.25-0.71), PCP years in practice (OR, 1.33 per 10 years in practice; 95% CI, 1.08-1.65 per 10 years in practice), and having nurse practitioner credentials (OR, 0.45; 95% CI, 0.17-0.98 compared with having physician credentials).

**Table 4.** Clinic and Primary Care Professional Characteristics

Characteristic	Clinics or professionals, No. (%)	Received ≥1 fundoscopic examination in 2019, No. (%)		Odds ratio (95% CI)		*P* value^a^
		No (n = 674)	Yes (n = 93)	Unadjusted	Adjusted	
**Clinic characteristics (n** **=** **28)**						
Located in a rural area	8 (28.6)	258 (38.3)	17 (18.3)	0.36 (0.20-0.61)	0.46 (0.18-1.17)	.11
Located in a primary care shortage area	6 (21.4)	252 (37.4)	19 (20.4)	0.43 (0.25-0.71)	0.59 (0.23-1.41)	.25
Located in a medically underserved area	6 (21.4)	314 (46.6)	35 (37.6)	0.69 (0.44-1.08)	NA	NA
No. of ophthalmologists per capita (county-level), median (IQR)	5 (4-20)	5 (4-20)	5 (5-20)	1.01 (0.99-1.03)	NA	NA
**PCP characteristics (n = 197)**						
PCP credential						
Physician	142 (72.1)	544 (80.7)	82 (88.2)	1 [Reference]	1 [Reference]	NA
Nurse practitioner	32 (16.2)	89 (13.2)	6 (6.5)	0.45 (0.17-0.98)	0.23 (0.04-0.79)	.049
Physician assistant	23 (11.7)	41 (6.1)	5 (5.4)	0.81 (0.27-1.93)	0.87 (0.28-2.18))	.78
PCP years in practice, median (IQR)	18 (9-28)	18 (9-28)	22 (19-28)	1.33 (1.08-1.65)^b^	1.26 (1.01-1.59)^b^	.04

Abbreviations: NA, not applicable; PCP, primary care professional.

^a^ Values are from the multivariable model, which included years in practice of PCP; PCP credential; rural clinic location; whether eye care professional examination was documented as up-to-date, documented as not up-to-date, or not documented; and whether the clinic location was in a primary care shortage area.

^b^ Per 10 years in practice.

The aforementioned variables were included in a multivariable model shown in Table 5. In the adjusted model, only PCP years in practice (adjusted OR [AOR], 1.26 per 10 years; 95% CI, 1.01-1.59; *P* = .04) and having nurse practitioner credentials (AOR, 0.23; 95% CI, 0.04-0.79 compared with having physician credentials, *P* = .049) were significant after adjustment for documentation of an up-to-date eye care professional examination by the patient’s PCP at least once during the year, clinic location in a rural area, and clinic location in a primary care shortage area.

**Table 5.** Factors Associated With PCP Performing Fundoscopy at Least Once in 2019

Factor	AOR (95% CI)	*P* value
PCP, years in practice (10-y increments)	1.26 (1.01-1.59)	.04
PCP credential		
Physician	1 [Reference]	NA
Nurse practitioner	0.23 (0.04-0.79)	.049
Physician assistant	0.87 (0.28-2.18)	.78
Rural clinic location	0.46 (0.18-1.17)	.11
Clinic location in primary care shortage area	0.59 (0.23-1.41)	.25
≥1 PCP documentation of eye care professional examination^a^		
Up-to-date	1 [Reference]	NA
Not up-to-date	0.56 (0.27-1.08)	.10
Not documented	0.97 (0.54-1.69)	.92

Abbreviations: AOR, adjusted odds ratio; NA, not applicable; PCP, primary care professional.

^a^ The eye care professional examination was considered to be up-to-date if the PCP documented it as up-to-date at any time during 2019.

In the encounter-level analysis, compared with attending a diabetes follow-up visit, attending an annual physical examination visit type was associated with 4.91 times greater odds of PCPs performing fundoscopy (95% CI, 2.57-9.38); new diabetes visit type (OR, 1.60; 95% CI, 0.20-13.03) was not associated with PCPs performing fundoscopy. Having a history of posterior eye disease was not associated with PCPs performing fundoscopy (OR, 0.77; 95% CI, 0.41-1.46).

## Discussion

In this retrospective review of electronic health records of patients with diabetes seen in a large primary care network, PCPs performed a documented fundoscopic examination at least once during the year for only 12.1% of patients. To our knowledge, this is the first study to report on clinical practice patterns of fundoscopic examination for DR screening in primary care. Our finding complements the existing literature reporting low rates of DR screening^2,4,15^: approximately 40% of patients in this study did not receive a screening examination from either an eye care professional or their PCP, suggesting that current primary care systems are insufficient to fill gaps in eye care for patients with diabetes.

We found no patient factors associated with PCPs’ performance of fundoscopy, including risk factors for DR, report of eye concerns at the visit, and relevant eye disease history. Rather, PCP and practice characteristics, including PCP years in practice and PCP credential, were associated with whether fundoscopy was performed. These findings suggest that PCP experience, training, and practice environment are more likely to be associated with PCPs’ DR screening practice patterns than patient risk factors for DR.

Our finding that all PCP documentations of fundoscopic examination results were normal, even though approximately 40% of the same patients were found to have disease when seen by an eye care professional, raises concerns about the utility of fundoscopy as a DR screening tool in primary care. Although prior studies have suggested that training may improve PCPs’ accuracy in DR screening,^6,7,16^ the cost-effectiveness of doing so at a population level needs to be compared with other population-based screening strategies, such as teleretinal imaging programs and artificial intelligence–based diagnostics. This study calls into question the practicality of DR screening using fundoscopy in typical primary care settings.

### Limitations

This study has limitations. One limitation is its use of data from electronic health records because documentation in electronic health records may not be comprehensive. Examination by an eye care professional was an imperfect surrogate for a criterion diagnosis because eye care professional examinations took place at different times and under different conditions than examinations performed by PCPs; however, eye care professional examination is the best surrogate available from electronic health record data. Causality cannot be established because of the retrospective nature of this study. Although the study included 28 different clinics, they were all affiliated with a single North Carolina health system, limiting generalizability. We also were not able to evaluate the length of time since a diagnosis of diabetes was made or whether referral to an eye care professional was made during primary care visits given the limitations of our data source.

## Conclusions

In this cohort study, fundoscopy was rarely performed and was not sensitive for detecting DR in primary care practice. Because rates of DR screening by eye care professionals remain low, research to explore and break down barriers to the implementation of effective primary care–based DR screening strategies, such as teleretinal imaging, is needed to prevent vision loss from DR.

## Notes

Supplement. **eTable.** List of *ICD-CM-9* and *ICD-CM-10* Codes Used to Identify Patients with Diabetes Mellitus
