# Supplementary material for: Practice Patterns of Fundoscopic Examination for Diabetic Retinopathy Screening in Primary Care
Source: JAMA Netw Open. 2022 Jun 27;5(6):e2218753. doi: 10.1001/jamanetworkopen.2022.18753 (PMC9237789; doi:10.1001/jamanetworkopen.2022.18753)
Supplement: Supplement. — eTable. List of ICD-CM-9 and ICD-CM-10 Codes Used to Identify Patients with Diabetes Mellitus [file jamanetwopen-e2218753-s001.pdf]

Supplementary Online Content

Song A, Lusk JB, Roh KM, et al. Practice patterns of fundoscopic examination for diabetic retinopathy screening in primary care. *JAMA Netw Open*. 2022;5(6):e2218753. doi:10.1001/jamanetworkopen.2022.18753

eTable. List of *ICD-CM-9* and *ICD-CM-10* Codes Used to Identify Patients with Diabetes Mellitus

This supplementary material has been provided by the authors to give readers additional information about their work.

© 2022 Song A et al. *JAMA Network Open*.

eTable. List of *ICD-CM-9* and *ICD-CM-10* Codes Used to Identify Patients with Diabetes Mellitus

List of ICD CM-9 and ICD CM-10 Codes Utilized to Identify Diabetes Mellitus in the Study
249.00, 249.01, 249.10, 249.11, 249.20, 249.21, 249.30, 249.31, 249.40, 249.41, 249.50, 249.51, 249.60, 249.61, 249.70, 249.71, 249.80, 249.81, 249.90, 249.91, 250, 250.0, 250.00, 250.01, 250.02, 250.03, 250.1, 250.10, 250.11, 250.12, 250.13, 250.2, 250.20, 250.21, 250.22, 250.23, 250.30, 250.31, 250.32, 250.33, 250.4, 250.40, 250.41, 250.42, 250.43, 250.5, 250.50, 250.51, 250.52, 250.53, 250.6, 250.60, 250.61, 250.62, 250.63, 250.7, 250.70, 250.71, 250.72, 250.73, 250.8, 250.80, 250.81, 250.82, 250.83, 250.9, 250.90, 250.91, 250.92, 250.93, 253.5, 357.2, 588.1, 648.00, 648.01, 648.02, 648.03, 648.04, 775.1, E08.00, E08.01, E08.10, E08.11, E08.21, E08.22, E08.29, E08.311, E08.319, E08.321, E08.3211, E08.3212, E08.3213, E08.3219, E08.329, E08.3291, E08.3292, E08.3293, E08.3299, E08.331, E08.3311, E08.3312, E08.3313, E08.3319, E08.3391, E08.3392, E08.3393, E08.3399, E08.3411, E08.3412, E08.3413, E08.3419, E08.3491, E08.3492, E08.3493, E08.3499, E08.351, E08.3511, E08.3512, E08.3513, E08.3519, E08.3521, E08.3522, E08.3523, E08.3529, E08.3531, E08.3532, E08.3533, E08.3541, E08.3542, E08.3543, E08.3551, E08.3552, E08.3553, E08.3559, E08.359, E08.3591, E08.3592, E08.3593, E08.3599, E08.36, E08.37X3, E08.39, E08.40, E08.41, E08.42, E08.43, E08.44, E08.49, E08.51, E08.52, E08.59, E08.610, E08.618, E08.620, E08.621, E08.622, E08.628, E08.630, E08.641, E08.649, E08.65, E08.69, E08.8, E08.9, E09.00, E09.10, E09.21, E09.22, E09.29, E09.311, E09.319, E09.3292, E09.3391, E09.3511, E09.3513, E09.3519, E09.3541, E09.3551, E09.3553, E09.359, E09.3592, E09.3593, E09.3599, E09.36, E09.39, E09.40, E09.41, E09.42, E09.43, E09.44, E09.49, E09.51, E09.52, E09.59, E09.610, E09.618, E09.621, E09.622, E09.628, E09.630, E09.649, E09.65, E09.69, E09.8, E09.9, E10.10, E10.11, E10.21, E10.22, E10.29, E10.311, E10.319, E10.321, E10.3211, E10.3212, E10.3213, E10.3219, E10.329, E10.3291, E10.3292, E10.3293, E10.3299, E10.331, E10.3311, E10.3312, E10.3313, E10.3319, E10.3391, E10.3392, E10.3393, E10.3399, E10.341, E10.3411, E10.3412, E10.3413, E10.3419, E10.349, E10.3491, E10.3492, E10.3493, E10.3499, E10.351, E10.3511, E10.3512, E10.3513, E10.3519, E10.3521, E10.3522, E10.3523, E10.3529, E10.3531, E10.3532, E10.3533, E10.3539, E10.3541, E10.3542, E10.3543, E10.3551, E10.3552, E10.3553, E10.3559, E10.359, E10.3591, E10.3592, E10.3593, E10.3599, E10.36, E10.37X3, E10.37X9, E10.39, E10.40, E10.41, E10.42, E10.43, E10.44, E10.49, E10.51, E10.52, E10.59, E10.610, E10.618, E10.620, E10.621, E10.622, E10.628, E10.630, E10.638, E10.641, E10.649, E10.65, E10.69, E10.8, E10.9, E11.00, E11.01, E11.10, E11.11, E11.21, E11.22, E11.29, E11.311, E11.319, E11.321, E11.3211, E11.3212, E11.3213, E11.3219, E11.329, E11.3291, E11.3292,

© 2022 Song A et al. *JAMA Network Open*.

E11.3293, E11.3299, E11.331, E11.3311, E11.3312, E11.3313, E11.3319, E11.339, E11.3391, E11.3392, E11.3393, E11.3399, E11.341, E11.3411, E11.3412, E11.3413, E11.3419, E11.349, E11.3491, E11.3492, E11.3493, E11.3499, E11.351, E11.3511, E11.3512, E11.3513, E11.3519, E11.3521, E11.3522, E11.3523, E11.3529, E11.3531, E11.3532, E11.3533, E11.3539, E11.3541, E11.3542, E11.3543, E11.3551, E11.3552, E11.3553, E11.3559, E11.359, E11.3591, E11.3592, E11.3593, E11.3599, E11.36, E11.37X1, E11.37X2, E11.37X3, E11.37X9, E11.39, E11.40, E11.41, E11.42, E11.43, E11.44, E11.49, E11.51, E11.52, E11.59, E11.610, E11.618, E11.620, E11.621, E11.622, E11.628, E11.630, E11.638, E11.641, E11.649, E11.65, E11.69, E11.8, E11.9, E13.00, E13.01, E13.10, E13.11, E13.21, E13.22, E13.29, E13.311, E13.319, E13.3213, E13.3219, E13.3292, E13.3293, E13.3299, E13.331, E13.3313, E13.3399, E13.341, E13.3413, E13.3419, E13.3493, E13.3499, E13.351, E13.3511, E13.3513, E13.3519, E13.3521, E13.3523, E13.3532, E13.3543, E13.3552, E13.3553, E13.3559, E13.359, E13.3591, E13.3592, E13.3593, E13.3599, E13.36, E13.39, E13.40, E13.41, E13.42, E13.43, E13.44, E13.49, E13.51, E13.52, E13.59, E13.610, E13.618, E13.620, E13.621, E13.622, E13.628, E13.630, E13.649, E13.65, E13.69, E13.8, E13.9, O24.011, O24.012, O24.013, O24.019, O24.02, O24.03, O24.111, O24.112, O24.113, O24.119, O24.12, O24.13, O24.311, O24.312, O24.313, O24.319, O24.32, O24.33, O24.811, O24.812, O24.813, O24.819, O24.82, O24.83

© 2022 Song A et al. *JAMA Network Open*.
